# Winning at all costs: a review of risk-taking behaviour and sporting injury from an occupational safety and health perspective

**DOI:** 10.1186/s40798-019-0189-9

**Published:** 2019-05-02

**Authors:** Yanbing Chen, Conor Buggy, Seamus Kelly

**Affiliations:** 1 Institute of Sport and Health, School of Public Health, Physiotherapy and Sports Science, University College Dublin, Dublin, Ireland; 2 Centre for Safety and Health at Work, School of Public Health, Physiotherapy and Sports Science, University College Dublin, Dublin, Ireland

**Keywords:** Occupational health, Athlete safety, Injury risk, Elite athletes, Safety management

## Abstract

Professional athletes involved in high-performance sport are at a high injury risk, which may lead to long-term health consequences. Professional athletes often expose themselves to risky behaviours, resulting in a higher acceptance level of occupational risk compared to other occupations. To date, many studies have focused on elite athletes’ specific injury prevention techniques. The objective of this narrative review is to (1) summarise elite athletes’ attitudes towards important occupational safety and health (OSH) practices, including injury reporting, medicine usage and personal protective equipment (PPE) usage, and (2) explore factors that may influence elite athletes’ injury awareness. If injury awareness were given a similar weighting in elite sports as in any other highly physical occupation, the potential benefits to elite athletes and their long-term health could be highly significant. This review identifies that most elite athletes are not aware that sporting injuries are occupational injuries requiring behaviours determined by OSH rules. All the 39 studies identified met the moderate methodological quality criteria according to the Mixed Methods Appraisal Tool (MMAT). The factors impeding athletes’ injury awareness from achieving occupational health standards are discussed from three safety management perspectives: organisational, societal and individual. This review contributes to a better understanding of how to build a positive safety culture, one that could reduce elite athletes’ injury rate and improve their long-term wellbeing. Further research is required to develop a quantitative measurement instrument to evaluate occupational health awareness in the sport context. Based on the papers reviewed, the study population was categorised as elite, professional, high-performance amateur and student-athletes.

## Key points

- The key factors influencing an elite athlete’s occupational safety and health (OSH) awareness have been neither evaluated nor adequately identified in research studies to date.
- Occupational risk communication should be improved by establishing a proactive injury prevention culture and identifying clear-cut responsibilities for key stakeholders within sport organisations.
- Future research could develop an instrument focused specifically on the unique sport setting, which would elicit the factors hindering the improvement of elite athletes’ OSH awareness.

## Background

In an article about concussion in professional rugby union, ex-Scotland representative player Rory Lamont comments that “a flagrant disregard for your own welfare almost seems a prerequisite for achieving success at the highest level” [1]. The pressure of sports competitiveness inspires elite athletes to “win at all costs” [2] and may lead them to neglect injuries and continue playing with pain [3–6], resulting in a higher occupational risk acceptance [5]. Sporting injuries limit an athlete’s preparation and subsequent performances, which can have both short-term [6] and long-term health impacts [7]. According to statistics reported in the UK, the overall injury risk in professional soccer is 1000 times higher compared to other high-risk occupations such as construction and mining [8, 9]. One potential reason for this is that non-sporting workplaces have adopted OSH management practices to reduce the risk to as low as reasonably practicable (ALARP) [10]. OSH management practices have been embedded into organisational management in international and national health and safety legislation [11–13] but not completely adopted in elite sport organisations [14]. The incorporation in elite sport of OSH practices to reduce risky behaviours could minimise the health impacts of inevitable sporting injuries in the long-term [15]. If injury awareness were given a similar weighting in elite sport as in any other highly physical occupation, the potential benefits to elite athletes and their long-term health and wellbeing could be highly significant.

To date, researchers have studied sporting injury from different perspectives. For example, of the studies reviewed, many focus on elite athletes’ injury-related knowledge or certain types of injury prevention techniques, such as ankle sprain prevention [16] and knee injury prevention [17]. While some studies focus on the relationship between injury risk and individual factors such as personality traits [18, 19] and BMI [20], other studies assess injury risk in elite sport from a general health and safety perspective [8, 14, 21]. By reviewing existing studies across the disciplines of both OSH and sporting injury, this narrative review provides an OSH insight into elite sport to (1) summarise elite athletes’ attitudes towards important OSH practices, including injury reporting, medicine usage and personal protective equipment (PPE) usage, and (2) explore factors that may influence elite athletes’ injury awareness from three safety management perspectives: organisational, societal and individual. This review contributes to a better understanding of how to build a positive OSH culture in sport, one that could reduce injury rate and improve elite athletes’ long-term wellbeing.

## Literature search and evaluation methodology

**Fig. 1:**
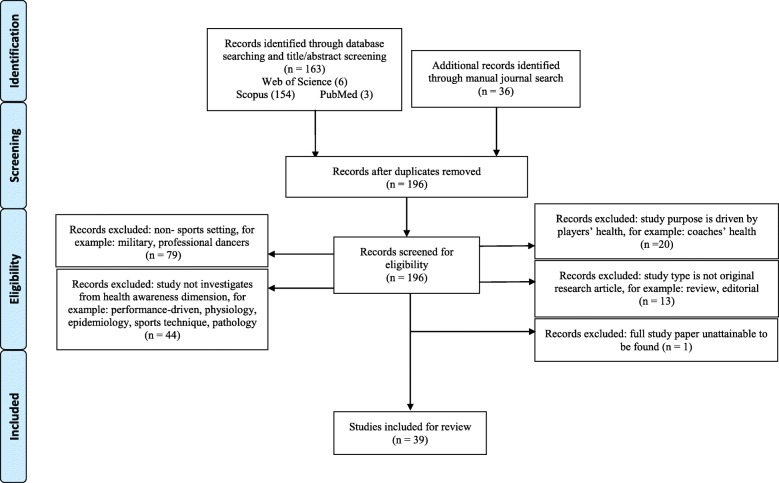
Flowchart diagram of search process

This paper adopts a narrative review method to transmit knowledge and information across the two disciplines [22]. The databases searched were PubMed, Scopus and Web of Science. The Boolean operators AND, OR and NOT were used to narrow or broaden the literature search. The combination of key terms searched are (“elite athletes” OR “professional players” OR “professional athletes” OR “elite players” OR “professional sports” OR “elite sports”) AND (“occupational safety” OR “occupational health” OR “occupational risk” OR “occupational health and safety”) AND (awareness OR perception). Because, considered occupational health terms in sports may not have been commonly used, articles identified by manual journal search were also included for a more comprehensive coverage. Since each database provides different facilities for paper screening, papers were generally screened by limiting publication date (2008–2018), article type (original research), English written, publication status (fully publicised) and text availability (full-paper available) in the searched databases. Explicitly, 47 books, 3 book series, 3 conference proceedings, 27 review papers, 1 conference paper, 1 short survey and 7 articles written in a language other than English (i.e. French, Spanish, German, Portuguese, Croatian, and Polish) were excluded. After limiting the publication date from 2008 to 2018, 163 documents were identified. Following a manual search, 36 papers were identified. One hundred ninety-six articles were identified after removing duplication. The first author reviewed and assessed all the 196 articles retrieved against criteria agreed by the other two authors to determine their inclusion in the final sample. On a case-by-case basis, we excluded a further 157 articles which met the key word criteria but following a review of the title and abstract, were not relevant to this study. In the first instance, we excluded 79 articles which focused on a non-sport setting (e.g. military). Furthermore, studies on dancing were not included in this review. In the second instance, we excluded 20 articles in which the health awareness of sport supporting staff is examined rather than that of athletes (e.g. coaches’ burnout, physiotherapists’ burnout or Paralympic leaders’ stress). Subsequently, 44 studies not focused on health awareness were also excluded (e.g. studies focused on the association between training load and sleep quality, studies focused on associations between common mental disorder symptoms and potential stressors or studies focused on associations between athlete burnout, insomnia, and polysomnographic indices among athletes). The abstract of the studies were reviewed if the information the study title provided was insufficient for screening. For example, studies such as *Mental and psychosocial health among current and former professional footballers* were excluded after reviewing the abstract, in which the association between the prevalence of mental health complaints and the psychosocial problems was examined rather than how players are aware of their mental health. In addition, 13 studies that did not meet the study type were excluded. We also excluded another one paper, the full text of which could not be located. Finally, 39 studies meeting the inclusion criteria were included. Figure 1 flowchart diagram indicates the overall search process. Table 1 presents the study information of the final included 39 papers after the inclusion and exclusion criteria screening process conducted. The study population of athletes was categorised as professional, elite, high-performance amateur, student athletes and mixed population as presented in Table 1.

**Table 1:** Study information

Study	Participants	Sample size	Player type	Methods	Awareness measured	Methods validity	Associations
Case-control study							
Barnard [23]	77 student athletes and 50 student non-athletes	127 Case group (athletes): 32 M 45F Control group (non-athletes): 13 M 37F	Student-athletes	Questionnaire	Mental illness	***	Athletes and non-athletes did not significantly differ in willingness to seek mental health treatment. Discrimination to mental illness: non-athletes > athletes. Willingness to seek psychological help: *F* > *M.*
Fedor and Gunstad [24]	382 college athletes and 230 college non-athletes	612 Case group (athletes): 228 M 154F Control group (non-athletes): 77 M 153F	Student-athletes	Questionnaire	Concussion	***	Concussion symptoms identification: athletes > non-athletes (*p* < 0.01)
Cohort study							
Kroshus et al. [25]	146 ice hockey players	146 M 6 teams	Elite	Questionnaire	Concussion	****	No statistically significant changes were observed in knowledge (*p* = 0.38), attitudes (*p* = 0.78) or perceived norms (*p* = 0.11). Inclination to play while concussed: before education > after education (lecture education: p = 0.02; email education *p* = 0.02).
Cluster-randomised controlled trial							
Cusimano et al. [26]	267 minor league hockey players	267; 10-year olds competitive, 106; 10-year olds recreational, 60; 14-year olds competitive, 54; 14-year olds recreational, 47.	Elite	Questionnaire	Brain injuries	**	Concussion knowledge: before video education < immediately after video education (p < 0.01). Concussion knowledge at 2 months: no significance between video and no-video groups (controlling for prior knowledge level, age and competitive level) (*p* = 0.52). Attitudes and behaviour scores at 2 months did not differ between groups *p* = 0.51.
McKay et al. [27]	31 female soccer teams, 29 coaches,258 players	Baseline: 47 coaches, 385 players Post-season: 29 coaches, 258 players	Elite	Questionnaire	Extremity injuries	****	Postseason: players > coaches considered “inadequate warm-up” as a risk factor for injury (*p* < 0.01). The belief that injuries are preventable: coaches > players answer “yes” (*p* = 0.00).
Grounded theory study							
Hachfeld et al. [28]	23 student-athletes	23 M	Student athletes	Focus group	Testicular cancer	***	Student athletes were more likely to perform testicular self-examination than the general student population and physical awareness is the core structural process that influenced the action.
Cross-sectional study							
Azodo et al. [29]	156 basketball players	156 124 M 32 F	Mixed	Questionnaire	Orofacial injuries	***	The prevalence of injury was not significantly associated with demography, category, competition and duration of participation (*p* = 0.26).
Berry et al. [30]	158 players at 10 institutions of the Central Collegiate Hockey Association	158 68 defensive, 90 offensive. Sex not indicated	Elite	Questionnaire	Orofacial injuries	***	No one specific factor affecting attitudes was identified. Negative attitudes towards mouthguard usage: defensive players > offensive players (*p* < 0.05).
Bhambhani et al. [31]	99 Paralympians with spinal cord injuries	99 85 M, 11 F 3 not indicated sex	Elite	Questionnaire	Autonomic dysreflexia	***	The awareness of the signs/symptoms and consequences of boosting was not associated with their education level (*p* = 0.58) or injury duration (*p* = 0.22).
Blank et al. [32]	883 junior athletes’ parents	883 409 M 474 F	Student-athletes	Questionnaire	Medicine use	***	Knowledge: Male parents > female parents; Parental sex did not demonstrate a significant influence on attitudes towards doping (p < 0.01).
Bloodgood et al. [33]	252 youth athletes and 300 parents	Parents: 90 M 210F Youth: 207 M 45F	Student-athletes	Questionnaire	Brain Injury	***	Agreed concussions are “a critical issue”: 13–15 years > 16–18 years (*p* < 0.05). Concussions are “a critical issue”: mothers> fathers (*p* < 0.05). Disagree “dumb for caring about concussions”: girls> boys (*p* < 0.05).
Broglio et al. [34]	727 soccer professionals	727 650 athletes 43 coaches 34 medical staff Sex not indicated	Professional	Questionnaire	Concussion	***	The following are reasons for not reporting concussions: Believe the injury was not serious (72.7%); not knowing it was a concussion (18.2%); not want the team down (4.5%); Believe concussions are part of the game (4.5%).
Brown et al. [35]	240 high school athletes (cross-country, volleyball, soccer, tennis, drill, cheer, colour guard, band, and swimming) and their 10 coaches	240 F athletes, 10 coaches	Student-athletes	Questionnaire	Female triad risk	***	Average triad knowledge score differed among teams (*p* = 0.01); triad awareness among athletes (average knowledge score was 2.79 ± 1.61 out of 8).
Chan et al. [36]	410 athletes from individual sports (athletics-track, athletics-field, badminton, gymnastics, swimming, and triathlon) and team sports (cricket, soccer, field hockey, basketball, rugby and water polo)	410 227 M 183 F	Elite	Questionnaire	Medicine use	****	When controlled motivation is low: autonomous motivation ↓ → doping intention ↑ (*p* < 0.01). When controlled motivation is high: no significant between autonomous motivation and doping intention (*p* = 0.57). When autonomous motivation was low: controlled motivation↓ → doping intention ↑ (*p* < 0.01); when autonomous motivation was low: no significant between controlled motivation and doping intention (*p* = 0.50).
Coffey et al. [37]	149 professional and semi-professional soccer players	149 M	Professional	Questionnaire	Concussion	***	Concussion report odds: defenders > other playing positions (*p* = 0.05).
Cournoyer and Tripp [38]	334 varsity high school soccer players	334 Sex not indicated	Student-athletes	Questionnaire	Concussion	**	No correlations were found between the method of education and the knowledge of symptoms or consequences of concussion (1 − *β* = 0.82).
Kerr et al. [39]	214 former NCAA collegiate athletes	214 140 M 74 F	Mixed	Questionnaire	Concussion	***	In low/noncontact sports: self-identified sports-related concussions non-disclosure: *M* > *F* (PR = 2.88).
Kuhl et al. [40]	94 equestrian riders	94 27 M, 67 F 64 amateurs 30 professionals	Mixed	Questionnaire	Concussion	**	Experience level did not influence the rates of concussion (*p* value not reported).
Kurowski et al. [41]	496 high school athletes	496 384 M 112F 212 American football 123 soccer 89 basketball 72 wrestling	Student-athletes	Questionnaire	Concussion	***	No association found between improved concussion knowledge and improved self-reported behaviours (*p* = 0.63); Age (*p* = 0.01) ↑ & female sex (*p* = 0.03) → concussion knowledge↑; Age (*p* = 0.01) ↓ & female sex (*p* = 0.00) & soccer participation (*p* = 0.02) → self-reported behaviours ↑.
Ma [42]	236 basketball players	236 M 77 professionals 159 semi-professionals	Mixed	Questionnaire	Orofacial injuries	**	The incidence of dental and oral injuries was related to the length of training time (*p* value not reported).
McCrea et al. [43]	1532 varsity soccer players from 20 high schools	1532 Sex not indicated	Student-athletes	Questionnaire	Concussion	***	No significant relationship found between a player’s prior concussion history and the likelihood of concussion reporting during the season.
Meyers et al. [44]	298 athletes in non-traditional non-NCAA sports (downhill skiing, martial arts, rock climbing, rodeo, skydiving and telemark skiing) and traditional NCAA sports (equestrian, golf, swimming/diving, tennis and track)	298 F 152 non-NCAA athletes 146 traditional NCAA athletes	Mixed	Questionnaire	Pain-coping	***	Women athletes pain-coping traits: non-traditional individual-sport activity < coach-structured traditional NCAA sports (Wilks’ *λ* F6,291 = 12.92; *p* = 0.00).
Miyashita et al. [45]	454 high school athletes	454 242 M 212 F	Student-athletes	Questionnaire	Concussion	**	Participants were asked if the importance of a game/event should dictate when they are allowed to return to play, and 50.9% stated “yes” with no difference between sexes (*p* = 0.10) or age (*p* = 0.19).
Muwonge et al. [46]	360 professional athletes (basketball, soccer, handball, rugby, athletics and cycling)	360 218 M 142 F	Professional	Questionnaire	Medicine use	****	Female athletes mean PEAS scores: with a prior doping history > without doping history (*p* = 0.10)
Norcross et al. [47]	66 soccer and basketball coaches from 15 high schools	66 coaches: 16 boys soccer 17 girls soccer 18 boys basketball 15 girls basketball	Student-athletes	Questionnaire	Lower extremity injury	***	Coaches’ injury prevention programs awareness: girls’ team > boys’ team (*p* = 0.00); Soccer > basketball (*p* = 0.05).
Onyeaso and Adegbesan [48]	42 coaches of secondary school athletes	42 25 M 17 F	Student-athletes	Questionnaire	Orofacial injuries	**	Statistically significant association (*p* < 0.05) was found between the sports and usage of mouthguards by the athletes as claimed by the coaches.
Overbye [49]	775 elite athletes from 40 sports	775 465 M 310 F	Elite	Questionnaire	Medicine use	***	Interests in anabolic-androgenic steroids use: M > F (*p* = 0.00); Speed and power sports athletes> motor-skill sport athletes (p = 0.02); Team sports athletes >motor-skill sport athlete (*p* = 0.08); Endurance sport athletes > motor-skill sport athletes (*p* = 0.15).
Register-Mihalik [50]	167 high school athletes	167 97 M 55 F	Student-athletes	Questionnaire	Concussion	***	No association found between increased athlete knowledge and attitude and prevalence of playing while experiencing concussion symptoms (*p* = 0.84).
Reuter and Short [51]	154 noncontact/limited-contact sports athletes	154 Swimming 27 M 18 FTrack 26 M 28 FBaseball 25 M	Elite	Questionnaire	Perceived risk of injury	**	Uncontrollable injury scores showed a significant difference between 3 sports (all about *p* = 0.00) with baseball players fearing the most risk and swimmers fearing the least. Risk of controllable injuries showed a significant difference between swimming and baseball (*p* = 0.01) with baseball players fearing the most risk and swimmers the least. Risk of upper body injury scores indicated a significant difference between track and swimming (p = 0.00) and track and baseball (p = 0.00). Swimmers reported the most fear of upper body injury while track athletes scored the lowest. Risk of re-injury scores indicated a significant difference between track and baseball (*p* = 0.00), and baseball and swimming (p = 0.00).
Shendell et al. [52]	1138 endurance athletes (full marathon, half marathon, and wheelchair athletes)	1138 499 M 639 F	Mixed	Questionnaire	Asthma	****	About 12.10% participants reported physician-diagnosed asthma; 84.6% correctly knew an asthma action plan can prevent hospitalizations; 18.0% reported they had an asthma action plan;24.8% had ever been asked to demonstrate medication use (controller and/or rescue inhaler) but only 2 people performed daily peak flow measurements.
Short et al. [53]	434 contact sports athletes	434 Hockey, 86 M 76 F Soccer. 32 M 32 F American football, 208 M	Elite	Questionnaire	Perceived risk of injury	**	Worry/concern↑ → probability of injury↑ (*p* < 0.01). Worry/concern ↑ → confidence in avoiding injury↓ (p < 0.01). Perceived probability of injury↑ → confidence in avoiding injury↓ (p < 0.01). Confidence in avoiding injury: M soccer previous injured< M hockey Previous injured (ES = 0.52). Confidence in avoiding injury: M soccer uninjured > M hockey uninjured (ES = 0.68). Confidence in avoiding injury: F uninjured > F previous injured (ES = 0.38. Perceived probability of injury: F previous injured > M previous injured (ES = 0.72). Confidence in avoiding injury: F soccer >F hockey (ES = 0.86). Worry/concern about injury: F hockey >F soccer (ES = 0.85). Worry/concern: M soccer> M hockey (ES = 0.25).
Shroyer and Stewart [54]	53 rural high school coaches	53 17 M 36 F	Student-athletes	Questionnaire	Concussion	**	13% of coaches knew and 48% did not know high school athletes take longer to recover from a concussion than do older athletes.
Sorkkila, Aunola and Ryba [55]	391 student-athletes from 6 upper secondary sport schools and their parents	391 student-athletes: 49% M 51% F 448 parents: 188 M 260 F	Student-athletes	Questionnaire	Burnout	***	The higher success expectations in sport: school burnout group > mild sport burnout group (*p* < 0.01); The higher success expectations in school: mild sport burnout group > school burnout group (*p* < 0.05).
Strotmeyer and Lystad [56]	175 amateur Muay Thai fighters	175 114 M 61 F	High-performance amateur	Questionnaire	General injuries	***	Muay Thai fighters perceived the risk of injury in their own sport to be average and significantly lower than that in other collision and contact sports (*p* < 0.01).
Tiwari et al. [57]	320 national and international level players (wrestling, karate judo, boxing, Wushu, fencing, taekwondo, hockey, canoeing and kayaking, rowing, sailing, horse riding, and shooting)	320 213 M 2017 F	Professional	Questionnaire	Orofacial injuries	**	Awareness and use of mouthguards: contact sports athletes > noncontact sports athletes (*p* = 0.00).
Tulunoglu and Oezbek [58]	274 semi-professional or amateur boxers and taekwondo players	274 174 M 100 F	Mixed	Questionnaire	Orofacial injuries	**	Mouthguard awareness: players with a dental trauma experience > players without a dental trauma experience (*p* = 0.00); Players with a facial trauma experience > players without a facial trauma experience (*p* = 0.01).
Therkorn and Shendell [59]	120 participants including college athletes, coaches and athlete parents/guardians	120 26 coaches 37 college athletes 57 athlete parents/guardians	Student-athletes	Questionnaire	Asthma	**	The percentage of correct responses by coaches to 5 asthma knowledge questions ranged from 12% to 88%.
Williams et al. [60]	26 professional soccer players	26 M	Professional	Questionnaire, interview	Concussion	****	The mean score on concussion knowledge was 16.4 ± 2.9 (range 11–22) and the attitude score was 59.6 ± 8.5 (range 41–71); The interview responses identified inconsistencies between the concussion knowledge/attitude and the intended behaviours, endorsing multiple concussion misconceptions, and revealed barriers to concussion reporting.
Zech and Wellmann [61]	139 professional and youth players	139 24 First Team players 18 U23 players 25 U19 players 17 U17 players 20 U16 players 35 U15 players Sex not indicated	Mixed	Questionnaire	General injuries	***	Perceptions on risk factors for injuries: athletes with previous injuries > athletes without previous injuries (fatigue: *p* = 0.04; previous injuries: *p* = 0.01; environment *p* = 0.00).

*M* male, *F* female, *U* under, > more than/higher than, < less than/ lower than, ↑ increase, ↓ decrease, & and, % per cent, *p p*-value, *PR* prevalence ratio, *ES* effect size, *NCAA* National Collegiate Athletic Association;

According to Mixed Methods Appraisal Tool (MMAT) [62], the score of study quality is presented using descriptors * (scores varying from 25% (*)—one criterion met—to 100% (****)—all criteria met);

Mixed: player type is mixed by professional and non-professional players as the study indicated.

### Inclusion criteria

The following are the inclusion criteria:

1. Qualitative or quantitative original research published in academic journals written in English
2. A study population who were currently or previously involved in *elite* sports (“rule-governed, structured, competitive gross movement characterised by physical strategy, prowess and chance” [63])
3. Studies that investigate awareness of sporting injuries or risk-taking behaviours

### Exclusion criteria

The following are the exclusion criteria:

1. Studies on dancing
2. Studies that focus purely on injury prevention skills or techniques and do not measure awareness (i.e. physiology, epidemiology, sports technique, pathology)
3. Articles reporting second-hand data only (i.e. review papers, editorials, letters, books, book chapters, conference papers, theses and other unpublished works)
4. Studies that did not focus on player health (i.e. studies focused on coaches’ health were excluded)
5. Studies where the full paper was unable to be retrieved

The quality and strength of the selected studies were graded according to the criteria of the Mixed Methods Appraisal Tool (MMAT) by the first author in consultation with the two co-authors. The MMAT can be used to evaluate qualitative, quantitative and mixed-methods studies, especially health-related studies [62]. The papers reviewed were categorised by study type: case-control study, cohort study, cluster-randomised controlled trial, grounded theory study and cross-sectional study. The data extracted from each identified study included study year, participants, sample size, research methods, awareness type (i.e. specific sporting injuries or risk-taking behaviours) measured and significant associations. The scores calculated using the MMAT are presented in Table 1 varying from * (one criterion met, rating of 25%) to **** (all criteria met, rating of 100%). Overall, the methodological quality of the 39 identified studies ranged from 50% to 100%. Among those, 12 studies were of moderate quality (rating of 50%) and 22 studies were of high quality (rating of 75%) and 5 studies were of excellent quality (rating of 100%).

## Results

After reviewing the papers included, the three most frequent themes were injury reporting, medicine usage and PPE usage. The results of the remaining studies on other health and safety issues have also been included in the table, though not discussed within the three themes.

### Sporting injury- concussion reporting awareness

Sporting injuries can be classified as occupational injuries because elite athletes are contracted or remunerated employees in sport organisations. Under the legislation in most developed countries [12, 13], employees are responsible for notifying their employer of occupational injuries. Accordingly, elite athletes should report their sporting injuries to the management staff as soon as possible. However, some elite athletes fail to report sporting injuries and consequently miss the time window for medical treatment [34]. From the papers reviewed, there are mainly four injury-reporting failures. First, elite athletes do not realise that they are actually injured [43]. Second, elite athletes do not think that their injuries are serious enough to report. Third, elite athletes do not disclose sporting injuries because of pressure from coaches, teammates, fans and parents [64]. Finally, elite athletes report the injury as regulated but the injury disclosure is then underestimated by the management staff [65].

Based on the ALARP standard [10], occupational injury reporting as a basic OSH management practice has not been adequately adopted within elite sport. In terms of the first two injury-reporting failures mentioned above, it is important for elite athletes to perceive injury symptoms and injury consequences. Of the 39 articles reviewed, seven studies examined injury symptom awareness and six studies examined injury consequence awareness: most of these studies focusing on brain injuries, especially concussion, are presented in Tables 2 and 3, respectively. This may because brain injuries are more difficult to detect when compared to limb injuries, which can hinder sport performance in a more obvious manner. Taking this into account, the findings in this section can only be discussed based on concussion. Most of the studies reviewed on concussion awareness have an overall high methodological quality.

**Table 2:** Professional Athletes’ Awareness of Injury Symptom Identification

Study	Awareness measured	Participants	Sex	Mean age (years)	Findings
Broglio et al. [34]	Concussion	650 soccer players, 43 coaches, 34 medical staff	Not indicated	16.8	10.0% athletes sustained a concussion in the past year and 62.0% of these injuries were not reported. Medical staff reported a heavy reliance on the clinical exam (92.0%) and athlete symptom reports (92.0%) to make the concussion diagnosis and return to play decision, with little use of neurocognitive (16.7%) or balance (0.0%) testing.
Cournoyer and Tripp [38]	Concussion	334 high school soccer players	Not indicated	16.3	Concussion symptom identified: headache (97.0%); dizziness (93.0%); confusion (90.0%); Loss of consciousness (81.0%); nausea or vomiting (53.0%); behaviour and personality change (40.0%); trouble falling asleep (36.0%); being more emotional (30.0%); being nervous or anxious (27.0%).
Fedor and Gunstad [24]	Concussion	382 college athletes and 230 college non-athletes	M/F	Case group: 19.6 Control group: 19.6	Student-athletes expected significantly more distractor (1.19 ± 1.05 vs. 0.84 ± 0.89), somatic (6.05 ± 1.76 vs. 5.30 ± 2.12), and cognitive (2.31 ± 0.90 vs. 2.01 ± 1.10) symptoms compared with controls. No significant differences emerged for emotional or sleep symptoms.
Kerr et al. [39]	Concussion	214 former collegiate athletes in NCAA	M/F	Not indicated	70.4% did not know when they had suffered a concussion.
McCrea et al. [43]	Concussion	1532 high-school soccer players	Not indicated	Not indicated	36.1% lacked awareness of probable concussion.
Miyashita et al. [45]	Concussion	454 high-school athletes	M/F	15.7	The number of athletes who reported at least 1 concussion history: before study session < after study session (*p* = 0.00).
Williams et al, [60]	Concussion	26 professional soccer players	M	59.6	80.8% athletes who were knocked unconscious would be taken to the emergency room. 80.8% managers would keep players with concussions out of games; 69.3% physiotherapists making return to play decisions regarding concussions. 38.5% athletes would play with a concussion during semi-final playoff games. 57.7% athletes would play through a headache resulting from a concussion.

*M* male, *F* female; *%* percent, < less than/ lower than, *p p*-value

**Table 3:** Professional athletes’ awareness level of injury consequences

Study	Awareness measured	Participants	Sex	Mean age (years)	Findings
Broglio et al. [34]	Concussion	650 soccer players, 43 coaches, 34 medical staff	Not indicated	16.8	Most soccer players did not feel that the injury was serious enough to report; 72.0% coaches understood that having a single concussion increases the risk of a second injury concussion risk
Cournoyer and Tripp [38]	Concussion	334 high-school soccer players	Not indicated	16.3	Possible concussion consequences correctly identified: Brain haemorrhage, coma, and death (60.0% to 70.0%); Early-onset dementia (64.0%); Early-onset Alzheimer disease (47.0%); Early-onset Parkinson disease (28.0%). Improperly identified: increased risk of blindness with age (50.0%) and increased risk of stroke (38.0%)
Kuhl et al. [40]	Concussion	94 equestrian riders	M/F	Not indicated	88.0% agreed or strongly agreed repeated head injuries could result in lasting impairments; 76.0% believed that concussions can increase brain injury; 27.0% believed that work or academics was likely to worsen concussion symptoms; 47.0% disagreed or strongly disagreed that concussion management should be more conservative for a child.
Ma [42]	Orofacial injuries	236 basketball players (77 professionals and 159 semi-professionals)	M	Not indicated	59% ranked the risk of orofacial and dental injury in basketball as medium.
McCrea et al. [43]	Concussion	1532 high-school soccer players	Not indicated	Not indicated	66.4% of the players would not report concussion because they did not think it was serious enough for medical attention.
Williams et al. [60]	Concussion	26 professional soccer players	M	59.6	96.0% indicated playing with a concussion may increase later life risk of “serious stuff” or “cognitive problems”, but 64.0% would continue to play when suffered a concussion.

*M* male, *F* female, *%* percent

Furthermore, an untreated brain injury may have grave health consequences [66–69], which is a serious occupational risk of which elite athletes should be aware. If elite athletes are unaware of injury consequences, they may not report the injury. For example, one study with a 47% response rate found a cohort of Italian soccer players did not feel that a concussion was serious enough to report and therefore went untreated [34]. Another study using a self-administrated survey collected data from high school football players based on retrospective recount of concussion found that numerous players continue playing before fully recovering from concussion [43]. The phenomenon that players returning to play before fully fit has also been found in youth hockey by direct game observation with retrospective surveys [70]. A failure in the translation and implementation of concussion return-to-play regulations was also found in rugby by examining whether players received return-to-play advice post-concussion and complied with it [71].

After a concussion, 52% of rugby players make their own decision to continue playing, while only 22% return after medical clearance [72]. The Pitch Side Concussion Assessment (PSCA) tool has been widely used [73], but after suffering a concussion elite players may deliberately cheat to pass the test in order to return to the field according to news reported [74] and previous review paper discussed [75]. Similarly, in non-contact sports like equestrian sports, concussion can equally lead to serious long-term effects [76–79] but many professional equestrian riders are not aware of the consequences. Almost half of the equestrian riders in Kuhl’s [40] study that received a self-reported concussion were likely to return to training or competing without seeking medical clearance. After experiencing concussion symptoms, more than 30% of riders thought that they could get back on a horse on the same day [40].

Concussion consequences could be minimised if both elite athletes and their supporting staff had better concussion-reporting awareness. This requires encouraging a culture of concussion disclosure in the organisation and adequate knowledge of concussion symptoms. As to the third injury-reporting failure, many studies found that the pressure from individuals or groups around elite athletes such as coaches, teammates, fans and parents could directly or indirectly impede injury-reporting behaviour [64]. These influences on injury reporting will be further discussed in the “Societal factors” section. In addition, while many elite athletes do successfully report their injuries, management staff such as clinicians may allow athletes to continue playing after a head impact based on their subjective interpretation of the concussion symptoms due to a lack of quantitative information [65, 80]. A lack of objective tools to diagnose concussion is no doubt an issue in this case. Nevertheless, if elite athletes and their supporting staff had a better OSH awareness to prioritise health and safety rather than sports performance, a more conservative and safer decision could be made.

### Awareness of the health risks of inappropriate medicine usage

All of the four studies reviewed on medicine usage awareness have high or excellent methodological quality. The main reason for the World Anti-Doping Agency (WADA) ban on the use of certain medicines is because of an actual or potential health risk to the athlete [81]. Many elite athletes take non-doping-classified medicines for enhancing athletic performance or treating injuries. From an OSH perspective, a control measure can increase the occupational risk if it is not appropriately managed. In this case, there will be a potential source of harm or adverse health effect on elite athletes if the medicine is taken improperly. Injured athletes who fail to report an injury may take medicine to mask pain so they can continue training and competing [82]. By examining urine sample of athletes in the Olympic Games in Sydney 2000, a study pointed to a dangerous overuse of nonsteroidal anti-inflammatory agents [83]. Blood sample measurements from athletes (*n* = 330) in the 2004 New Zealand Ironman triathlon identified the prevalence of non-steroidal anti-inflammatory drugs (NSAID) was 30% [84]. Another study [85] has identified a high rate of non-prescribed use of NSAID consumption among triathletes from 23 different countries, but it was not specified how the questionnaires were distributed and collected. Among younger athletes, a study [86] reported nearly one of seven high school football players used NSAIDs daily, according to data from self-administered questionnaires. However, the incidence might be under-reported considering coaches distributed the questionnaires which may lead to bias. These studies indicate elite athletes frequently take incorrect doses for extended periods and are not aware of the potentially deleterious adverse effects. Elite athletes have also been shown to use NSAIDs the day before competing for pain prevention [85, 86], such as for delayed-onset muscle soreness [87] as a “prophylactic pain treatment” [88]. Medicine usage for pain prevention can be found in various sports such as American football [86], soccer [88], marathon running [89] and triathlon [84].

Injured athletes can reduce rehabilitation time by using NSAIDs, but if they return to intensive training too soon they may be at risk of overuse injuries and inappropriate biomechanical stress [90, 91]. Some types of sports involving extensive and strenuous use of limbs and muscle groups may cause chronic tendinopathies and inflammation, in which conditions professional athletes may take NSAIDs with medical instruction [92]. Though permitted by WADA, elite athletes should not take non-doping classified medicines without medical supervision [93] because of the risk of the side effects [94–96]. Many athletes are still unaware that inappropriate medicine use can adversely affect their wellbeing in the long-term.

### Personal protective equipment (PPE) usage awareness

Among the seven papers reviewed relating to PPE, most papers have a moderate methodological quality. PPE is the least preferred solution in the control measures offered by OSH [97], but it plays an important role in various industries. A retrospective cohort study [98] in Washington State regarding road safety found that un-helmeted motorcyclists have nearly four times the risk of critical head injuries compared to helmeted riders despite incomplete associations of crashes to hospitalisations which may result in underestimates of the incidence of injuries. A review study [99] has indicated that helmets can prevent pedal cyclists from sustaining head and facial injuries by reviewing five well-conducted case control studies selected since no randomised controlled trials were found. An ecologic study of protective equipment and injury [100] found that wearing PPE can protect elite athletes from injury in collision in two contact sports. One of the contact sport in this study is American football, of which the data were reported by athletic trainers required by the on-going surveillance system of National Collegiate Athletic Association (NCAA) whereas the other contact sport is rugby with data collected from player self-reported for only one season. Examples can be seen in the categories of PPE associated with injury prevention available to elite athletes. This includes equipment for head and orofacial protection (e.g. helmets [99] and mouthguards [101]), extremity and joint protection (e.g. ankle braces and knee pads [102]) and genitourinary organ protection (e.g. athletic cup). PPE usage is thus crucial for some elite sports since other control measures [97] may not always be adequate or applicable in the sports context.

In combat sports, various types of protective gear have proved effective. Through repeatability tests by a spring driven linear impactor (Punch machine), one study [103] identified the important role of Association Internationale de Boxe Amateur (AIBA) headguards in reducing the risk of concussion and superficial injury in boxing competition and training. By reviewing 24 articles of level I or II evidence for prognostic studies, one systematic review paper [104] found that the implementation of full facial protection can reduce the risk of overall head and facial injuries in ice hockey compared with partial facial protection. This study also found that partial facial protection still offers more risk reduction than no facial protection.

In junior ice hockey, a 3-year prospective cohort observational analysis of elite players from 10 teams in 10 cities across 5 states played in the same league governed by the same rules and referees demonstrated how both full and partial facial protection can significantly reduce eye and face injuries without increasing neck injuries and concussions. Furthermore, the eye injury risk was 4.7 times greater for players with no protection compared with those wearing partial protection [105].

A retrospective analysis of data collected from 1833 American footballers between 2005 and 2010 from 8 collegiate teams found that a helmet is the primary item of equipment used to protect a player from head-related injury and may reduce the risk of sustaining a concussion [106]. Similar results have been observed by another three-year prospective cohort study with 2141 high school American football players [107]. It has been reported that the implementation of National Operating Committee on Standards for Athletic Equipment (NOCSAE) helmet standards in American football have resulted in an 88% decrease in serious head injury: this resulted in an average of only 0.51/100,000 players during the 2002–2006 seasons compared to that an estimated average 4.25/100,000 players in the 1964–68 era [108]. However, the effect of helmet use on brain injury risk in some sports is still contentious according to a systematic review conducted by Benson [109].

In a general working environment, positive safety culture includes the enforcement of appropriate PPE usage [110]. In comparison, the usage of PPE in sports is frequently optional or poorly implemented. Given the opportunity for nonresponse bias of a self-administered questionnaire by mail, Hawn [111] found that mouthguard use among NCAA ice hockey players in competition is not consistently enforced as it should be according to the sport’s regulations. Recently, World Rugby has adopted the regulation requiring players of all levels to wear adequate equipment such as mouthguards during training and competition [112]. This practice was strongly recommended by the Irish Rugby Football Union (IRFU) from the 2015/16 season but there is limited data on the effect of this regulation’s implementation to date.

Most of the reviewed studies on PPE usage (shown in Table 4) reported on athletes’ awareness of PPE, as opposed to its effectiveness. Mouthguards may help to reduce the probability of oral trauma, brain injuries, cerebral haemorrhage and possibly even death [113]. Orofacial injuries often result in life-long sequelae requiring expensive follow-up treatment which could be significantly reduced or avoided by the use of a mouthguard [113–117]. However, a high level of ignorance regarding mouthguard use is reported by analysing survey data collected from 1478 soccer and 1192 rugby players at high schools in Japan [118]. In a relatively recent study [42] in China, using an attitude survey after an epidemiological survey, 59% of basketball athletes investigated regarded basketball as a medium-to-high-risk sport, but few of them thought it necessary to wear a mouthguard. Considering the samples (*n* = 236) were all recruited from Chinese Basketball Association (CBA) based in Beijing, players in less developed cities could potentially report a lower awareness on mouthguard use. The reasons for not using a mouthguard included ignorance, non-availability, non-affordability, discomfort [29, 30] and their hindrance to verbal communication [42]. Participants in Berry’s study [30] tended to modify mouthguards to make them more comfortable ignoring the potential impact on their effectiveness. Similar to other control measures [97], the usage of PPE may create or contribute to occupational hazards for athletes, such as heat stress, physical injury and psychological pressure. From an OSH perspective, these issues must be acknowledged and confronted by athletes during PPE usage training [42].

**Table 4:** Professional athletes’ awareness of PPE use

Study	Awareness measured	Sports	Sex	Mean age (years)	Findings
Ma [42]	Mouthguard	Basketball	M	Not indicated	Awareness was high (80.1%), but the usage rate was low.
Azodo et al. [29]	Mouthguard	Basketball	M/F	23.1	There was a high prevalence of orofacial injuries and a low awareness of mouthguard use.
Berry et al. [30]	Mouthguard	Hockey	Not indicated	21.0	13.3% of players wore mouthguards most of the time during games; 3.8% wore mouthguards most of the time during practices.
Onyeaso and Adegbesan [48]	Mouthguard	Soccer, judo, boxing, hockey	M/F	38.1	81.0% coaches believed mouthguard should be worn at all times – during practice sessions and competitions; 19.0% coaches would prefer the use only during competitions.
Kuhl et al. [40]	ASTM/SEI-approved helmet	Equestrian	M/F	Not indicated	58.0% of riders strongly agreed on the use of helmets when jumping.
Tiwari et al. [57]	Mouthguard	Wrestling, karate judo, boxing, Wushu, fencing, taekwondo, hockey, canoeing and kayaking, rowing, sailing, horse riding, shooting	M/F	Not indicated	51.5% athletes were aware of mouthguards, but only 21.0% wore them.
Tulunoglu and Oezbek [58]	Mouthguard	Boxing, taekwondo	M/F	Not indicated	83.2% of participants knew the importance of using mouthguards.

*M* male, *F* female, % percent, *ASTM/SEI* American Society for Testing and Materials/Safety Equipment Institute

## Discussion

The factors hindering *elite* athletes’ occupational health awareness are interrelated. To demonstrate these factors in a clear way, this review categorised them into three different types: organisational safety management factors, societal factors and individual factors.

### Organisational Safety management factors

An organisation’s OSH system will not be effective without a positive safety culture [119]. Safety culture consists of shared values, attitudes, perceptions, and beliefs that drive decisions and behaviours regarding safety [120], and can be manifested by human workplace behaviours in the organisation as a part of organisational culture [121, 122]. When a positive safety management culture that is fostered by all in an organisation, positive behavioural change that is measurable ensues, this would also potentially be equally true in any sporting organisation that places an emphasis on safety.

#### Safety culture in sport

As Yau [123] reported with regard to the declining accident trend in the construction sector in Hong Kong, safety culture is the key to building a safer workplace. From an OSH perspective, a culture’s continual improvement cycle involves policy, organising, planning and implementation, evaluation and action for improvement [124]. Nevertheless, competitive sporting culture can conflict with the promotion of a positive safe working environment for elite athletes that includes OSH as a core aspect of risk management. Since mid-Victorian times, the ethics of muscular Christianity (e.g. teamwork, manliness, the moral and physical beauty of athleticism, discipline and self-sacrifice) underpinned rugby development within British and Irish public schools [125]. Sport cultures generally encourages athletes to keep competing regardless of pain [126, 127], to return quickly after injury [128] and to normalise injuries from a young age [129]. Sport-related identity, masculinity and risk-taking are components of the emerging portrait of a “toxic jock” [130], which may indicate an elevated risk of health-compromising behaviours [131]. Many elite athletes tend to be tough-minded and consequently they accept greater risk of injury than other people might. Though the culture may vary within different sports, the attitudes towards pain and injury are not confined to one sport or one cohort of athletes. In non-contact sports such as equestrian sports, riders are still encouraged to get back onto their horses immediately following a fall [40]. Furthermore, Paralympic athletes with spinal injuries intentionally induce autonomic dysreflexia for better performance, ignoring its risk [31].

Leadership plays a vital role in influencing the development of safety culture. In OSH management, senior managers in the human resources department are charged with “designing, fostering and nurturing” safety culture [132]. In a sport context, coaching staff can foster positive safety culture by encouraging injury-reporting habits through formal or informal means [25]. Likewise, leadership can destroy safety culture if it is not properly implemented [133]. For example, some studies found that coaches may exhibit a lack of injury identification knowledge [34] and compound the long-term impacts of injuries by neglecting injury prevention programmes [47].

#### Occupational risk communication

The communication between employer and employee is an important aspect of organisational safety management. Nevertheless, even if employers and management have adequate information, they cannot promote safety culture without effective communication.

First, employers are responsible for informing employees of occupational hazards that they may face in the workplace [12]. Like new employees, new elite athletes should be educated about the categories of playing conditions, hazardous sport-related factors, associated health effects and injury prevention techniques. A negative example in sports is that of the former American football players who sued the National Football League (NFL) for $1 billion for misrepresenting the long-term health impacts associated with on-field head injuries [134]. However, even if a sport organisation has an accurate understanding of the value of injury prevention techniques, the management team (such as coaching staff and medical staff) may fail to communicate this knowledge effectively to elite athletes [27]. Previous studies argued that there is no one-size-fits-all programme contributing to a better knowledge or understanding of injury consequences to elite athletes [38, 135]. Communication issues include not only access to safety knowledge but also the organisation’s preparedness and willingness to implement safety programmes [136]. Thus, it is necessary to customise the intervention programmes for each specific sport [137, 138]. Unfortunately, the effectiveness of such intervention programmes may not have a lasting impact. For example, Cusimano [139] demonstrated that the brain injury knowledge of elite athletes can be immediately improved through video education; however, this method lost its effectiveness after just 2 months.

Second, the management team should not only be a source of information in health risk communication but also recipients of such information. Elite athletes could take advantage of the internet to proactively share health-related learning materials via social media platforms. One such example is reducing substance abuse, where educational programmes have been successful [140]. However, the validity of online health-related resources that have a measurable impact on knowledge, awareness and consequent behaviour are a potentially contentious issue [141] that team management should be acutely aware of. Moreover, players should be given opportunities to communicate issues with their management team; otherwise, medical staff have limited chance to share their expertise with players on subjects such as injury management [142]. Effective health risk communication also depends on whether employees feel free to openly discuss safety issues within the organisation [143]. Open communication and frequent interactions are organisational features that differentiate companies with low injury rates from those with high injury rates [144]. Employees’ adequate OSH engagement is strongly promoted in general occupations [145], which suggests that employees’ opinions should always be considered. The aims of employee participation in instigating change are to raise awareness of non-specialists in addition to reaching consensus regarding the decisions, actions or policies aimed at managing and controlling risks [146]. Similarly in sport, communication between coaches and athletes has the potential to promote or undermine the ethics of playing while injured [147]. Therefore, elite players are key stakeholders when developing policies and strategies to address health and safety issues. Consequently, elite players’ voices and opinions should be included and may gradually impact managerial decision-making on OSH-related issues. Thereafter, the culture in sport organisation may be gradually changed to a more player welfare-centred approach in a similar manner to employees in non-sport occupations.

Third, occupational risk assessment is recognised as an integral part of successful OSH management [148]. The workplace is found to be much safer when the OSH-related practices are evaluated and feedback is given [149]. The purpose of carrying out a risk assessment is to determine whether the level of risk arising from workplace activities is acceptable, or whether more needs to be done to control or reduce risk. According to the Tolerability of Risk Framework [10], the UK Health and Safety Executive (HSE) expects that controls in place must, at a minimum, achieve the standards of relevant good practice precautions irrespective of specific risk estimates [150]. In most sports organisations, the acceptable level of occupational risk is higher than that in other industries. Thus, sports organisations may put greater emphasis on measures to control risk impacts rather than eliminating risks. This is an acceptable strategy for occupational risk management at the start of a risk assessment process as control measures evolve they should aim for risk elimination rather than control. However, a specific ALARP standard should be considered for risk level communication when occupational risk assessment is undertaken in sports organisations.

### Societal factors

Societal factors, ranging from the surrounding social referents (e.g. teammates and family) to the mass media, can shape an individual’s perception of health and safety over time. The most direct influences include the coach [29, 72], family and friends who are key referents in improving an athlete’s injury awareness [151].

Elite athletes exist in an environment of complex networks with various health hazards, which may influence injury disclosure behaviour [39] or drug-taking behaviour [152]. For instance, according to the UK’s online Drug Information Database (DID), 10% of UK professional athletes had contact with illicit drugs in both 2006 and 2007 [153]. The environment (inclusive of social interactions) may adversely affect elite athletes’ perception of health and safety, particularly younger athletes [154]. The attitudes surrounding social referents can be reflected in, or impact on, the behaviour of the elite athlete as they observe behavioural change in others they can increase their own awareness and consequently adapt and change their own behaviour, in essence a form of peer learning [39]. Kotarba [155] found that the “Sportsnet” [156] enabled athletes to share information on how to camouflage injuries that may threaten their active playing status. In addition, factors in the athletes environment such as coach approachability may also affect OSH management practices such as injury reporting [157]. Teammates’ or coaches’ beliefs and experiences (including risk-taking behaviour) may also affect elite athletes’ risk perception more than their own experiences [27]. For example, Kroshus [158] found that when collegiate athletes were pressured to continue playing after being concussed, they were more unlikely to reveal their symptoms. In Sye et al.’s study [72], 76% of rugby players believed that a teammate who had suffered from a brain injury would stay on during a game. Sye et al. [72] also reported that 151 elite athletes believed a concussed player on their team had continued playing due to pressure. Management and coaches may improve their own status (e.g. financial, commercial, status-related or career-related) by encouraging their athletes to make personal sacrifices “for the good of the team”. Therefore, responsibilities between athletes and staff are needed to be transparent in order to minimise the interference from personal interests.

The family plays an important role around *an* elite athlete. For example, mothers may be more likely than fathers to regard brain injury as a “critical issue” and are better at distinguishing concussion symptoms than fathers [159]. This may be due to the differing levels of interest in health issues exhibited by men as opposed to women and the way they view sports injuries [160, 161]. It is important for stakeholders such as parents to be aware of the long-term risks of injuries. The education of players’ family on injury consequences could be an effective approach, because family members may in turn encourage players’ injury disclosure by urging them to seek medical service.

Sport-related policies have an indirect but inevitable impact on the elite athlete’s OSH attitude. For instance, American college rugby has an injury rate three times higher than American football due to the lack of regulations stipulating the use of PPE [100]. After the National Football League implemented the “crown-of-the-helmet rule”, weekly reported brain injury injuries among defensive players reduced by 32%. However, weekly reported lower extremity injuries among offensive players increased by 34% [162], suggesting a change in tackling tactics in response to the rule change. In Azodo et al.’s study [29], basketball players suggested that mouthguard-wearing rate would be improved during the game if mouthguard usage was made compulsory. Therefore, sport-related policies may need to be updated and translated from OSH legislation in general to the specific sports context.

From the social dimension, the mass media can reinforce the image of the sport person by providing visual cues to audiences thus contributing to its role of “televised sports manhood formula” [163]. Some audiences prefer seeing sport violence that emphasises masculine hegemony [164] and this can, in turn, shape elite athletes’ behaviour. Even in female sport, a macho masculine-defined culture can be identified in the attitudes towards pain and injury [165]. The competitive culture driven by wider societal expectations of elite sport has become entrenched as one primary aspect of its own organisational culture. However, this situation can be augmented by adopting practices that engender a positive OSH culture, particularly in the processes relating to communication and consultation. Cusimano [139] deduced that long-term exposure to educational opportunities through coaches, parents and the media can make a bigger difference than short-term educational programmes. The active involvement of athletes’ families in the OSH process is essential to enhance their long-term wellbeing [166, 167]. It is essential for researchers to focus more on specific groups of elite athletes and their social milieu [162] to improve athletes’ OSH awareness multi-dimensionally.

### Individual factors

This section reviews the manner in which individual differences in terms of physical factors, personal experience and self-expectation influence elite athletes’ OSH awareness. Physical factors such as age, gender and ethnicity have been investigated regarding their influence on OSH awareness. These can be found in the “Associations” column in Table 1. Although physical factors are specific to the individual, a better understanding of them can contribute to effective interventions catering to different groups of elite athletes.

#### Injury experience

Attitudes are formed not only by social norms but also by personal experience [168, 169]. For example, elite athletes’ injury experience can be assimilated into their cognition, which may change their OSH attitude. Elite athletes with an injury history display more fear of injury than those who have never been injured [170]. For example, a rugby player who has incurred a previous shoulder injury may not be confident to take on tackles despite being fully recovered [171]. However, the fear of injury can protect athletes from returning to competitive action before they are fully recovered, which reduces the possibility of re-injury. This could prompt an athlete to seek OSH guidance or take measures to avoid injury. Players could improve their OSH awareness based on their injury experience. For example, in Ma’s study [42], only one of the basketball players was found using a mouthguard after a dental injury that dislocated his two incisors. Short [53] found that female soccer players with a history of injury reported greater risk perceptions than their uninjured peers. The reoccurrence of previous injuries can influence athletes’ occupational risk assessment and improve their awareness.

Athletes with greater playing experience in competitive leagues usually possessed enhanced injury knowledge [38]. Nevertheless, as playing experience increases, players are actually less likely to follow OSH management practices. For instance, McKay [27] found that the longer coaches and elite athletes are involved in soccer, the less likely they are to be willing to undertake the FIFA 11+ programme. McKay deduced that experienced elite athletes are so confident in their injury prevention techniques that they may undervalue the risk of injury during competitive games. This phenomenon exemplifies the fact that an athlete’s experience can influence his or her injury risk estimation.

#### Self-expectation of sporting performance

Self-expectation here refers to an individual elite athlete’s expectation of his or her sporting performance. Many elite athletes are willing to do whatever it takes to win, even sacrificing their health in order to leave a positive image while displaying toughness in order to earn peer group admiration and national recognition but also, importantly, financial rewards [6]. Athletes performing at a lower-level may be more eager to compete despite injury to gain respect and status by overconforming to the organisational and societal expectations placed upon elite sport [172–174]. Elite athletes may not report concussions because they do not want to stop playing or disappoint their teammates [43]. In team sports, a short period away from playing or training can have significant consequences for both the individual and the team [175]. The individual elite athlete may lose his or her position in the team, lose reputation due to missing training or lose earnings. Meanwhile, the team loses a potentially vital athlete that may impact team performance. As Rory Lamont, a retired rugby player who has cheated on concussion tests while playing the game, stated [1], “There is nothing more glorious than to show your teammates, coaches and fans that you are willing to put your body on the line for the cause”. He then explains that rugby culture is viewed as a bravado and self-sacrifice type culture where showing pain is a sign of mental weakness and may contribute to players losing the respect of teammates and coaches. However, the player also indicates this culture may have serious influence when it comes to concussion.

In Kerr’s et al. study [39], 67.6% professional athletes did not report injury because they did not want to be withheld from future game or practice. This study supports the view that self-expectation influences their injury-reporting behaviour, especially in countries where success at the university level of high-performance sport can facilitate an entry into a professional sporting career. This is validated by the fact that there is a practice among some professional rugby players to cheat on their return-to-play protocol after suffering brain injury by falsifying their cognitive tests for detecting brain injury [74, 75]. It is essential for the OSH philosophy to prevail by aiming at improving elite athletes’ attitudes towards both current safety at work and long-term health which may, in turn, lead to the protection of their wellbeing.

There are some limitations associated with the current review. First, studies meeting inclusion criteria cover widely from awareness of disease, burnout, injury, medicine usage and PPE usage, but this review focuses on injury reporting, medicine usage and PPE usage, the most frequent three themes for discussion as aforementioned. Second, considering the quality and heterogeneity of the studies reviewed, it is impossible to clearly conclude how athletes are aware of their health and safety from a quantitative data synthesis.

## Conclusion

In the studies reviewed, of seven studies on concussion symptom awareness, five studies reported on inadequate concussion symptom knowledge or awareness; of six studies examined injury consequence awareness, four papers reported inadequate concussion consequence awareness; and of seven studies on papers PPE, three studies reported inadequate mouthguard use. Since most of the included studies on sporting injury focused on concussion, the main discussion was based on concussion evidence, a topic which requires future research. Because OSH is a relative alien term in the sport literature the core purpose of this narrative review is to bridge the gap between the academic fields of OSH and sport.

The studies reviewed revealed that most elite athletes’ injury awareness does not meet the basic requirements of OSH standards. Existing injury prevention programmes focus more on techniques rather than consistently raising awareness. From an OSH perspective, risk communication practices should be improved in the sport context by establishing a proactive injury prevention culture, identifying clear-cut responsibilities between athletes and staff. In addition, factors influencing elite athletes’ OSH awareness have not been quantitatively measured since no such instrument has so far been devised. Arguably the development of an instrument or research toolkit linked to a conceptual framework is required to elicit the identification of the principal factors that could influence elite athletes from reaching appropriate levels of OSH awareness. Subsequently, OSH or welfare remedial programmes could be devised with the intention of improving awareness, enhancing involvement, increasing reporting and developing a safer sporting environment overall for the long-term wellbeing of athletes during and post-elite career.

## Abbreviations

AIBA: Association Internationale De Boxe Amateur
ALARP: As low as reasonably practicable
BMI: Body mass index
CBA: Chinese Basketball Association
DID: Drug Information Database
FIFA: Fédération Internationale de Football Association
HSE: Health and Safety Executive
IRFU: Irish Rugby Football Union
MMAT: Mixed Methods Appraisal Tool
NCAA: National Collegiate Athletic Association
NFL: National Football League
NOCSAE: National Operating Committee on Standards for Athletic Equipment
NSAID: Nonsteroidal anti-inflammatory drugs
OSH: Occupational safety and health
PPE: Personal protective equipment
PSCA: Pitch side concussion assessment
WADA: World Anti-Doping Agency
